# Can the Policy of National Urban Agglomeration Improve Economic and Environmental Gains? Evidence from Quasi-Natural Experiments with 280 Cities in China

**DOI:** 10.3390/ijerph19137596

**Published:** 2022-06-21

**Authors:** Fanchao Kong, Hongkai Zhang, Xiangyan Meng, Shuai Li, Jia Liu

**Affiliations:** 1 School of Statistics and Mathematics, Shandong University of Finance and Economics, Jinan 250014, China; k_fanchao@sdufe.edu.cn (F.K.); 202214062@mail.sdufe.edu.cn (H.Z.); 212214050@mail.sdufe.edu.cn (S.L.); 2 School of Insurance, Shandong University of Finance and Economics, Jinan 250014, China; 20197362@sdufe.edu.cn

**Keywords:** national urban agglomeration, economic growth, environmental pollution, green technology innovation, industrial structure

## Abstract

Urban agglomerations are an important symbol in the development of modernization. In this paper, we utilize the National Urban Agglomeration (NUA) policy as a quasi-natural experiment in the Chinese context. Adopting data from 280 cities from 2005 to 2019 as research samples, we use difference-in-differences (DID) and spatial difference-in-differences models (SDID) to examine the effect and mechanism of the implementation of the NUA policy on economic development and environmental pollution in China. The result shows that the NUA policy can achieve urban economic and environmental gains, which still holds after the robustness test. The heterogeneity analysis showed that the effects of the NUA policy are more evident in large and medium-sized cities. The curbing effect of the NUA policy on pollution emissions is apparent in the eastern region. Considering spatial heterogeneity, the expected economic and environmental benefits of the NUA policy are partially borne out. In addition to the green technology innovation, the NUA policy also influences regional economic development and environmental pollution through industrial agglomeration and the upgrading of industrial structures, respectively. It is essential to strengthen regional cooperation and establish the development concept of community interests between cities.

## 1. Introduction

Since the concept of urban agglomeration was introduced, many scholars have conducted numerous researches on the definition [1,2,3], morphological development [4], spatial organization [5,6], distribution pattern [7], and spatial evolution [2] of urban agglomerations. Urban agglomeration is a group of cities with compact spatial organization and close economic ties, generally with one or more mega-cities as the core and three or more large cities as constituent units, formed within a specific geographical area and relying on well-developed transport and communication networks [8]. Agglomeration benefits are vital drivers of economic growth and prosperity in large cities [9]. Labor productivity and wage premiums are better in densely populated and larger cities [10]. However, despite the higher productivity of larger cities, city size has not been the key to the growth of welfare in recent years [11]. Metropolitan functions rely more on inter-city network embeddedness than on city size [12]. In particular, various types of spatial organizations have been established in many regions, such as urban agglomerations [13], high-tech development zones [14], metropolitan areas [15], and university knowledge parks [16]. Of these forms, the urban agglomerations are among the most important in order to enable countries or regions to participate effectively in the global economy, process information and allocate capital [17]. The development experience of developed economies shows that dense areas represented by urban agglomerations can effectively promote regional development through knowledge spillover and industrial clustering [13]. The Great Lakes Urban Agglomeration in North America, the Atlantic Coastal Urban Agglomeration in the Northeast of the United States, the Pacific Coast Agglomeration in Japan and the British Urban Agglomeration are good examples. Competition between regions is increasingly concentrated on the level of combined power of urban agglomeration. Regional planning policy has become an effective way to reduce inter-regional disparities and promote coordinated economic and environmental development [18].

Over the past 40 years, China has carried out a number of regional adjustment policies at different stages of development. For example, the “Reform and Opening Up” policy was implemented in China in 1978, focusing on the establishment of special economic zones in the eastern coastal areas to attract foreign investment and develop the productive forces of society. The “Western Development” policy was implemented by the State Council of China in 1999 with the objective of narrowing the economic gap between the western region (comprising 12 provinces or autonomous regions) and the eastern coastal region. The “Rise of Central China” was approved in 2006 to explicitly build the national grain production base, the energy and raw material base, and the comprehensive transportation hub in the central region (including six provinces) in order to promote common development. The “One Belt, One Road” policy was introduced in 2013 to strengthen economic ties among countries along the route and to create demand and employment. This progression of policy implementation over a span of four decades suggests that national development planning has become an effective means of promoting China’s regional economic and social development [19,20,21].

The term urban agglomeration first appeared in the outline of China’s 11th Five-Year Plan in 2006. Since then, the National New Urbanization Plan (2014–2020) and the 13th Five-Year Plan (2016–2020) have explicitly proposed urban agglomeration as the main form of promoting the new urbanization since then. During this period, China’s State Council has approved 10 national urban agglomerations. “The 14th Five-Year Plan (2021–2025)” states that it is necessary to accelerate the integrated development of urban agglomerations to form the “two horizontal and three vertical” urbanization pattern. According to the China Urban Agglomerations Development Report in 2019, the 19 urban agglomerations generated more than 85% of the country’s GDP with an area of about 25%. The urbanization rate reached over 61% in these regions. Nonetheless, for a long time, under the fiscal decentralization system in China, local governments have played a dominant role in the process of regional development [22]. Due to the race to increase GDP and seek political advancement, protectionism of local officials and rent-seeking behavior aggravate regional market fragmentation [23], distorting market resource allocation, environmental pollution, and other problems [24]. As an important tool for modernization in China, can the implementation of the National Urban Agglomeration (NUA) policy bring about coordinated development of the economy and environment? Accurate evaluation of the effects of the NUA policy has become a hot issue of concern for government agencies and academics.

The NUA policy is formulated and approved by the Central Government and the State Council of China. The NUA refers to a spatially compact group of cities formed within a specific geographical area, centered on a large city and relying on a developed infrastructure network [8]. In 2015, the State Council officially approved the Yangtze River Central River City Agglomeration Development Plan, which became the first cross-regional city agglomeration approved by the State, and the remaining 10 national urban agglomerations were also approved in turn. Despite the close economic ties and social exchanges between the central cities and their neighboring cities before the approval, there are still outstanding problems such as the continuing widening of regional disparities, high costs of decentralization and obvious shortcomings in industrial collaboration [24]. In particular, large differences still exist between cities in terms of market access, administrative barriers, business environment and enterprise subsidies. For example, before the approval of the Chengdu-Chongqing City Agglomeration, Guang’an City in Sichuan Province was actively undertaking the transfer of automotive and electronic information industries from Chongqing City, but the policy standards in Sichuan and Chongqing were not uniform, and enterprises transferred from Chongqing to Guang’an City could not enjoy preferential subsidies, which directly affected the enthusiasm of Chongqing enterprises to settle in Guang’an.

The objective of the NUA policy is to explore a coordinated model of inter-city factor market management in order to promote coordinated regional development. To achieve these goals, each national-level urban agglomeration introduced corresponding specific measures after being approved. For example, the Yangtze River Delta Urban Agglomeration has established a joint organization for collaborative development, and it has established the Shanghai-Suzhou-Dafeng Industrial Cluster, the Zhangjiang National Independent Innovation Demonstration Zone and the Minhang National Demonstration Zone for the Transformation of Scientific and Technological Achievements and promoted the optimal layout of industrial chains. The Central Yangtze Urban Agglomeration has issued a series of documents, including the Action Plan for Collaborative Development and the Key Issues for Cooperation in Building 2020, and has established the “Four Cities, One Policy” talent system, shared atmospheric monitoring data, and promoted demonstration projects for the integrated application of industrial internet.

In addition, the approval of national-level urban agglomerations has enhanced the stringency and coordination of related policies. For example, to achieve joint prevention and control of air pollution, the Beijing-Tianjin-Hebei urban agglomeration has jointly implemented air pollution control actions for four consecutive years. The cities have signed the Framework Agreement on Joint Prevention and Control of Water Pollution Incidents in Cross-Provincial Watersheds and the Cooperation Agreement on Joint Prevention and Control of Hazardous Waste in North China in order to deal with cross-regional environmental pollution problems and illegal cases. The Yangtze River Delta urban agglomeration has formulated the Work Plan for Emergency Response to Cross-border Environmental Pollution Incidents and the Rules for Implementing the Action Plan for the Prevention and Control of Air Pollution in the Yangtze River Delta Region and jointly formulated special treatment schemes for the Yangtze River Estuary and Hangzhou Bay. Along with the implementation of the NUA policy, a series of related action plans have been introduced in various regions. Due to the different factor endowments, the schemes introduced in each urban agglomeration are different, and the transmission paths of the NUA policy effects may be diverse. The question of whether the NUA policy will deliver a double dividend is unclear, and it is, therefore, worth exploring whether and how it will affect economic growth and environmental pollution.

The reasons for using China as our setting for our empirical study are as follows. First, cities have increasingly become the center of human production and life. Urban agglomerations are an important symbol of the improved level of modernization. As the second-largest economy in the world, unlike developed economies such as the United States, China is still in the transition phase from the centrally planned to a market economy [23]. China must learn from the advanced experience of the first modernization transition in developed countries, such as labor transfer and forming a unified factor market. Furthermore, China must face the new situation of the second modernization transition, such as the information technology revolution and the regional ecological issues. In the context of China’s transition from the “administrative economy” to “economic integration”, it is critical to scientifically assess the economic and environmental effects of the NUA policy, especially for emerging economies. Second, as an old Chinese saying goes, “It is impossible to have a good plan for the present without any long-term strategy; it is impossible to handle the current situation without any overall planning.” Planning policy has become an essential tool for macroeconomic regulation by the Chinese government [22]. “The Five-Year Plan for National Economic and Social Development (the Five-Year Plan)” is the most important public policy in China’s policy system and is an essential expression of China’s institutional advantages and development experience. China has drawn up and implemented fourteen consecutive Five-Year Plans, which have played an irreplaceable role in guiding development, allocating public resources and achieving strategic goals. China’s HDI (Human Development Index) has risen from 0.41 in 1978 to 0.758 in 2018. According to the World Bank’s international poverty standards, 740 million people have been lifted out of poverty in China, accounting for around 70% of global poverty reduction. The 13th Five-Year Plan (2016–2020), which has already been carried out, and the 14th Five-Year Plan (2021–2025), which is currently being implemented, focus on the NUA policy as an important spatial vehicle for promoting coordinated development of the economy and environment. China’s policy system provides an ideal condition for testing the effects of the NUA policy.

Our study’s marginal contribution is summarized as follows. First, existing studies on regional integration typically use the price approach to measure the degree of regional integration rather than focusing on regional integration policy. In contrast, this paper uses the NUA policy as a quasi-natural experiment in the Chinese scenario to explore whether the NUA policy can deliver the dual dividend of promoting economic development and reducing environmental pollution. It can provide meaningful international lessons for other emerging countries. Second, we examine the heterogeneity of the NUA policy at the regional and scale levels. Third, this paper examines the transmission mechanism of the NUA policy, which is not abundantly addressed in the literature. We specifically explore the mechanisms of the NUA policy on economic growth and environmental pollution based on the mediating effect model, providing empirical experience for further understanding of the role of the NUA policy.

The rest of the paper proceeds as follows. Section 2 develops the research hypotheses. Section 3 describes the data, variables and methodology. Section 4 presents the empirical results. Section 5 concludes the paper.

## 2. Literature Review and Research Hypotheses

Urban development planning involves a complex relationship between economic growth, environmental protection and social equity in cities [25]. Many kinds of literature have focused on the impact effects of regional integration, which fall into two main areas. On the one hand, it has been found in the literature that urban integration can enhance economic productivity by accelerating factor mobility [26], promoting industrial upgrading [27], and strengthening regional cooperation [28], among others. Meijers et al. [29] examined whether the urban integration consisting of the polycentric urban region (PUR) in Europe affects the performance of PUR and found that functional and institutional integration of PURs positively affects the economic performance of PURs. He et al. [30] pointed out that the spatial organization of the Chang-Zhu-Tan urban agglomeration has a marked concentration trend, and the agglomeration effect has an important role in its integrated development process. On the other hand, urban integration enhances the use of environmental resources and thus reduces total regional pollution emissions [31]. Using inter-provincial panel data for China from 1995 to 2012 as a sample for the study, Li and Lin [32] found that regional integration has a significant positive influence on the energy and CO_2_ emissions performance. He et al. [33] found that China’s regional economic integration gradually increased from 2002 to 2011, contributing to the marginal abatement cost of CO_2_. Xiao et al. [34] found that regional integration within cities improves governance capabilities and facilitates the transfer of energy-intensive industries, which can reduce carbon emissions. He and Lu [35] examined the influence of regional integration on water pollution in the Yangtze River Economic Zone and showed that regional integration has a significant inhibitory effect on transboundary water pollution. In addition, urban regional integration buffers the role of environmental regulation [36], which can cause a decline in the regional environmental quality [37]. In summary, unlike urbanization, an urban agglomeration is no longer a single city but an inter-city agglomeration. The literature has studied the relationship between regional integration and energy, carbon emissions, the environment and economic growth, but there is no consensus on the findings. Although the urban agglomeration policy has been in place for many years, most studies have only focused on individual city clusters or economic zones, and most have used the price approach to measure the degree of regional integration rather than focusing on regional integration policy. Most of the literature does not focus on the changes brought about by the implementation of the NUA policy (e.g., market integration, removal of administrative barriers, free movement of factors), which are the focus of this paper.

The development of urban agglomerations in China has a heavily government-led background. In terms of the selection process, each national urban agglomeration contains at least one central city (e.g., provincial capitals, municipalities directly under the central government) that acts as a leader in the region. From a review of publicly available government documents, the selection of cities is based on factors such as their geographical location, topography, transportation, culture, and industrial structure. For example, in the Guanzhong urban agglomeration, Linfen is geographically 302 km from Xi’an, which is greater than its distance to Zhengzhou (243 km). Therefore, why is it not included in ZhongYuan urban agglomeration? The reason is that the Linfen Basin is in the same zone as the Guanzhong Plain, separated from it only by the Yellow River. Although Linfen is closer to Zhengzhou, these are separated by the Taihang Mountains. In addition, Linfen and Guanzhong have strong similarities in culture and customs. National urban agglomerations are planned by the State Council and are not randomly assigned. We regarded the implementation of the NUA policy as a quasi-natural experiment. We further developed the Propensity Score Matching—Difference-in-Differences (PSM-DID) model based on the baseline regression, matching cities in the treatment group with all cities in the untreated group, and were able to demonstrate that the variability in the grouping of subjects is not too great when using the DID model for policy evaluation. In practice, the NUA policy is not just a spatial regrouping of cities but more of a package of actions resulting from the implementation of the policy, involving tax incentives, factor mobility, financial support and market thresholds. This is the reason why this article tests the effects of the NUA policy.

**Figure 1:**
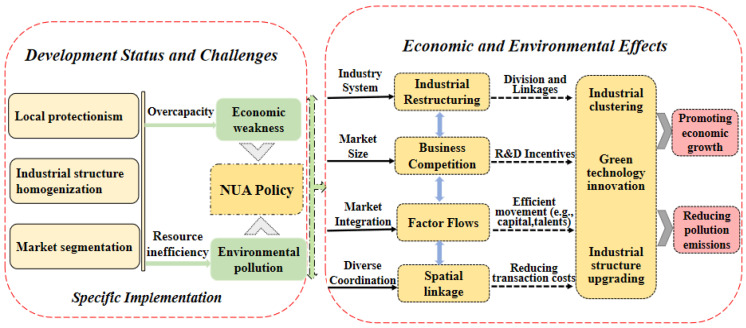
Mechanistic transmission diagram of the NUA policy and economic growth and environmental pollution.

The NUA policy reduces the degree of market segmentation and facilitates the circulation of production factors between cities through market integration, industry system, market size and diverse coordination, with implications for economic growth and environmental change. As shown in Figure 1, we focus on a regional integration policy and examine the economic and environmental effects of the NUA policy separately.

At the factor allocation level, the center-periphery model and the free capital model in the new economic geography explain how regional integration shapes the spatial distribution of economic activity based on factor mobility [38]. Regional integration facilitates market competition and promotes the free flow of innovation factors (e.g., capital, resources, talents, technology, knowledge, and information) [26]. It is guided by the market competition mechanism to make the endowment structure between cities a dynamic change, achieving an optimal combination in a larger space [39]. In the monopolistic competition model, the degree of market competition depends on the market size [40]. Regional integration has increased the size of the market, providing firms with more incentives to invest in the development of new products [29]. The efficient allocation of production factors can enhance the coupling between the industrial system and the innovation system among cities, leading to a positive convergence effect, thus helping to stimulate technological innovation and economic growth [27]. On the other hand, widespread market segmentation is a typical fact of China’s regional economy [22]. Local governments will break down barriers to market entry under integrated development, which can facilitate the development of the industrial division of the labor system within an urban agglomeration [41]. It will bring about regional industrial agglomeration [21,42]. In addition, based on the theory of circular cumulative causality, the industrial linkages, spatial overflow and input sharing generated by the integrated development can effectively accelerate the process of inter-city knowledge transfer and innovation diffusion, which enhances the efficiency of economic growth [19]. Thus, we propose two hypotheses.

**Hypothesis** **1** **(H1).** *The NUA policy has a significant positive effect on urban economic growth.*

**Hypothesis** **2** **(H2).** *The economic effect of the NUA policy can be delivered through green technology innovation and industrial agglomeration.*

Urban agglomeration integration not only stimulates factor mobility and achieves economies of scale [26], it also influences urban pollutant emissions through energy consumption and green technologies [14]. Firstly, local protectionism can lead to the convergence of regional industrial structures, which may result in overcapacity and low resource utilization [22]. Regional development planning can strengthen the relationship between local governments and reduce internal administrative barriers [23], integrating energy markets (e.g., establishing the regional pollution emission trading market) and improving the efficiency of using environmental factors [32,43]. In this context, regional integration brings specialization according to comparative advantages [39]. It facilitates market integration and the transfer of polluting firms [34] and promotes upgrading industrial structures (e.g., cooperating to establish industrial parks, industrial development zones, etc.) [14]. Furthermore, it can improve regional resource utilization, reduce the cost-sharing of polluting facilities and generate economies of scale in pollution control. As such, it is thereby incentivizing enterprises to reduce emissions [44]. Second, it has been shown that the diffusion of technological progress and management experience can be limited by regional market segmentation [45]. With the implementation of the NUA policy, regional market integration has been strengthened. Market competition motivates enterprises to innovate green technologies and adopt more environmentally friendly production techniques [46]. In addition, market integration can speed up the diffusion and application of energy-saving and emission reduction technologies and decrease the transaction costs of environmental protection technologies, improving the efficiency of energy factors and reducing pollution emissions. Thus, we propose the following additional hypotheses.

**Hypothesis** **3** **(H3).** *The NUA policy has a significant negative effect on urban pollution emissions.*

**Hypothesis** **4** **(H4).** *The environmental effect of the NUA policy can be delivered through green technology innovation and industrial structure upgrading.*

## 3. Materials and Methods

### 3.1. Sample Selection and Data Sources

The regional and temporal variations in implementing the NUA policy provide an opportunity for a DID analysis. Since 2015, China’s State Council has approved a total of 12 national urban agglomerations, namely: Northern Bay, Chengdu-Chongqing, Guanzhong Plain, Ha-Chang, Hubao-Egyu, Beijing-Tianjin-Hebei, Lanxi, South Central Liaoning, Yangtze River Delta, Mid-Yangtze River, Central Plains, and the Guangdong-Hong Kong-Macao Greater Bay Area. Considering that the Guangdong-Hong Kong-Macao Greater Bay Area was just approved in 2019, the remaining eleven urban agglomerations were selected for this paper due to the sample period and data availability constraints. In our study, the sample consisted of 280 cities in China. The list of specific city groups is given in Appendix A.

To ensure the quality of the sample data, the study sample was screened in the following manner: (1) we excluded cities with changes in administrative boundaries (e.g., Sansha, Haidong) and cities with serious data missing (e.g., Bijie, Tongren, Lhasa) in the period 2005–2019; and (2) we excluded areas with different social regimes (Macau, Hong Kong, and Taiwan). We finally selected 280 cities in China from 2005 to 2019 for our study. The data used in our research are drawn from several sources. Information on the timing of the approval of the NUA policy and the cities included was obtained from the website of the Central People’s Government of the People’s Republic of China. The remaining data were collected from the China City Statistical Yearbook from 2005 to 2019. In addition, taking 2005 as the base period, all price-specific data were adjusted to remove the influence of price fluctuations.

### 3.2. Selection of Variables

#### 3.2.1. Dependent Variables

The dependent variables include urban economic development (per capita gross domestic product (PCGDP)) and environmental pollution (SO_2_), measured by PCGDP (logarithm) and per capita sulfur dioxide emissions, respectively. In the robustness testing section, we also use employment (Employment), per capita smoke (Smoke) and per capita wastewater (Wastewater) in place of the above variables. We chose the per capita emissions of pollutants (logarithm) as a proxy for the environmental variables.

#### 3.2.2. Independent Variable

In our study, the sample consisted of 280 cities in China. Specifically, the sample is divided into two groups: the treatment group comprising 155 cities designated as NUA cities and the untreated group comprising 125 non-NUA cities. We set the dummy variable NUA_i_ depending on whether the city belongs to the NUA or not. NUA_i_ indicates city i’s NUA status, i.e., NUA_i_ = 1 if city I is an NUA city and NUA_i_ = 0 if city i is the non-TCZ city. We set the dummy variable Time_t_ according to when the city was approved. Time_t_ indicates the post-treatment period, i.e., Time_t_ = 1 when the city was approved as an NUA city and in subsequent years and Time_t_ = 0 otherwise. The core explanatory variable in our study is the NUA × Time, an interaction term of the dummy variables, equaling 1 after city i was approved as an NUA city, otherwise 0.

#### 3.2.3. Control Variables

Following previous studies, specifically [33,35], the control variables include consumer demand (Con), measured as the ratio of total retail sales of consumer goods to GDP; government demand (Gov), measured as the share of local government expenditure in GDP; governance intervention (GI), measured by the ratio of fiscal expenditure to fiscal revenue; the level of human capital (Hum), measured by the number of people employed in the city (taken as a logarithm); industrial structure (Ind), measured by the ratio of secondary sector value added to GDP; financial development (Fin), measured by the ratio of total deposits and loans of urban financial institutions to GDP; the share of fixed asset investment (Inv), measured by the ratio of the amount of fixed asset investment to GDP. In addition, we have dummy variables for industry and year in the regression model. The descriptive statistics for the variables are shown in Table 1.

### 3.3. Model Specification

To test hypotheses, we can then compare environmental pollution and economic development in NUA cities before and after implementing the NUA policy with the corresponding change in non-NUA cities over the same period. Following previous studies [47,48], the DID estimation model in our study is as follows:

y_it_ = β_0_ + β_1_DID_it_ + ∑γ_j_x_it_ + μ_i_ + η_t_ + ε_it_ (1)

where y_it_ is the measurement of dependent variables, including environmental pollution (SO_2_) and economic development (PCGDP), in city i at year t; β_0_ is a constant term; DID_it_ denotes the interaction term of the dummy variables (NUA_i_ × Time_t_), the coefficient β_1_ indicates the marginal effect of the NUA policy on economic development and environmental pollution; x denotes the matrix-vector of the control variables mentioned above; μ_it_ denotes the city fixed effect, η_it_ denotes year fixed effects, representing yearly factors common to all cities, such as industrial policy, monetary policy, macro uncertainty, etc., and ε_it_ denotes the random error term.

It is noted that the grouping of samples in natural experiments is a completely random “natural event” and can be regarded as random. In contrast, the selection of samples in quasi-natural experiments is often artificial, making it impossible to assign subjects in a completely random way. Although quasi-natural experiments relax to some extent, the assumption is that there are no fundamental differences between experimental and control groups, and they also require that the variability in the grouping of subjects is not too great when using the DID model for policy evaluation [49]. Thus, to check the robustness of the baseline regression model estimations, we also used PSM-DID, a placebo test, dynamic effect analysis and substitution of variables as the comparison.

Furthermore, referring to previous studies [50,51], we adopted the SDID model to identify the spatial spillover effects of the NUA policy on both NUA-designated cities (treated regions) and neighboring non-NUA-designated cities (untreated regions). The SDID model is set up as follows.

y_it_ = β_0_ + β_1_DID_it_ + ∑γ_j_x_it_ + β_2_W_T,T_D_it_ + β_3_W_NT,T_D_it_ + ∑β_j_Wx_it_ + μ_i_ + η_t_ + ε_it_ (2)

where W denotes the first-order adjacency weight matrix. β_2_ and β_3_ are the spatial coefficients of W_T,T_D_it_ and W_NT,T_D_it_, respectively, in which the former represents the spatial spillover effects on NUA-designated cities, the latter represents the spatial spillover effects on non-NUA-designated cities, and the β_j_ denote the spatial coefficients of x_it_. The other parameters are consistent with Equation (1).

## 4. Results

### 4.1. Baseline Regressive Results

We conducted the DID estimation model, controlling for year and individual fixed effects, to examine the NUA policy on economic growth and environmental pollution. Table 2 reports the regression results for Equation (1). The coefficients of DID are positive and significant at the 5% level in columns (1) and (2) when employing PCGDP as the dependent variable, indicating that the NUA policy positively affects urban economic growth. The results suggest that cities approved as NUA can achieve relatively faster economic growth in integrated regional development from economic actions generated by the NUA policy, supporting hypothesis H1. The coefficients of DID are negative and significant at the 1% level in columns (4) and (5) when employing SO_2_ as the dependent variable, indicating that the NUA policy has a negative effect on SO_2_ emissions. Compared to cities that have not been approved as NUA, the results suggest that cities that have been approved as NUA can decrease urban SO_2_ emissions, supporting hypothesis H3. These groups achieve environmental gains from environmental actions generated by NUA policy. Overall, the NUA policy has effectively promoted economic growth while curbing the emission of environmental pollutants.

### 4.2. PSM-DID

In an ideal natural experiment, the cities in the treated and untreated groups should conform to the random selection process. To test the robustness of the model results, referring to previous studies [51,52], we relax the assumption of individual randomness and apply the PSM-DID method for estimation. In the following way, we first use the control variables as matching variables and calculate the propensity score for each city using the logistic model and match the scores. Next, we estimate the impact of the NUA policy on economic development and environmental pollution using the DID method. The estimated results are shown in columns (3) and (6) in Table 2. The coefficients of DID were 0.024 and −0.122, respectively, and significant at the 5% level in columns (3) and (6), indicating that the NUA policy can significantly enhance urban economic development and reduce pollutant emissions. This finding is generally consistent with the baseline model, i.e., the conclusion that NUA policy can achieve urban economic and environmental gains for their cities is robust.

### 4.3. Placebo Test

The baseline model estimation findings may be influenced by the implementation of other national policies, leading to biased conclusions. To further test the robustness of the model results, this paper conducts the placebo test. We advanced the implementation of the NUA policy by 1, 2 and 3 years, respectively, and the corresponding estimation results are shown in Table 3. The coefficients of DID were 0.012, 0.001 and 0.007 in columns (1)–(3), and −0.093, −0.075 and −0.073 in columns (4)–(6), respectively, and were insignificant at 5%, indicating that the NUA policy fails to significantly affect urban economic growth and environmental pollution after advancing the implementation time by 1, 2, and 3 years, respectively.

**Figure 2:**
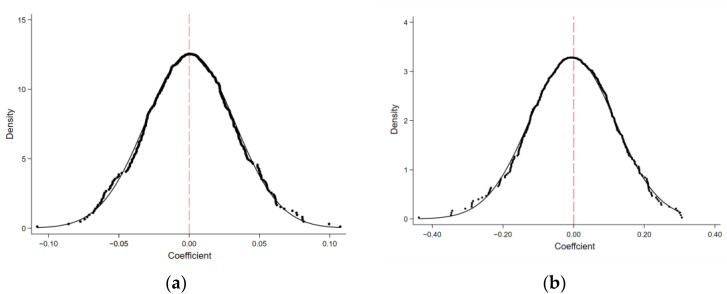
Placebo test. (**a**) economic growth. (**b**) environmental pollutant. Notes: the dotted line shows the cumulative distribution density of the coefficients is from 500 simulations randomly, and the solid line shows the normal distribution.

In addition, to test whether the baseline model results were due to unobserved factors, referring to the research of Li et al. [53], we also conducted a placebo test by randomly assigning pilot cities. The procedure was as follows: firstly, we randomly selected some of the cities as the treatment group and then assigned each of them a random year as the implementation time of the policy to obtain the cross-sectional term DID. Secondly, we collected 500 random samples and respectively ran baseline regressions according to Equation (1) and plotted the distribution of the 500 spurious estimated coefficients, as shown in Figure 2. If the estimated coefficients of DID under the random sampling experimental are distributed around 0, then the baseline regression results are not influenced by omitted variables or random factors. This implies that the economic and environmental effects we are concerned with are indeed brought about by the NUA policy. In Figure 2a,b, the coefficients of DID in the placebo test are clustered around zero, which is significantly different from the actual coefficients of DID (from columns (2) and (5) of Table 2). Thus, we can largely exclude the influence of other random factors on the baseline regression model.

### 4.4. Replacement of the Dependent Variable

We also use employment, smoke (dust) and wastewater in place of the above variables. As shown in Table 4, the coefficient of DID is significantly positive at the 5% level in column (1) when employment is the dependent variable. The coefficients of DID are negative and significant at the 10% level in columns (2) and (3) when employing smoke (dust) and wastewater as the dependent variables. The NUA policy has a positive effect on employment and a negative effect on urban smoke (dust) and wastewater emissions, suggesting that the findings of the baseline model still hold after replacing the dependent variables.

### 4.5. Endogeneity Test

The DID model may be able to solve the endogeneity problem through differencing, but it cannot address the sample selection problem. Therefore, we address reverse causality and potential endogeneity by applying the instrumental variables approach (IV) with two-stage regression estimation.

In the course of their historical development, the historic cities were metropolises integrating politics, economy and culture, which were interconnected with the surrounding areas in terms of transport, cooperation and exchange, folklore, and human relations. Practice has shown that there is a close link between the central city of the modern urban agglomeration and whether it is a historical capital or not [54,55]. There is insufficient empirical evidence to suggest that there are direct links between economic growth or environmental pollution in modern cities and those in historical capitals. Therefore, we use the interaction term (dummy variable measured by whether it is a historical capital multiplied by the DID) as an instrumental variable for the NUA policy. Based on the Wikipedia entry, we manually collated data on China’s ancient capitals through the ages and then matched them with modern city names, finding that a total of 59 prefecture-level cities overlapped. Table 5 lists the results of the regression model estimates. In the first-stage regression, the estimated coefficients of the instrumental variables are significant regardless of whether the dependent variable is economic growth or environmental pollution, and their corresponding F-values are all greater than 10, indicating that there is no weak instrumental variable problem in the model. In the second-stage regressions, the estimated coefficients on the core explanatory variable DID are all significant at the 1% level, and their corresponding signs remain consistent with the previous benchmark regressions, suggesting that the NUA policy effects still hold after accounting for endogeneity.

### 4.6. Heterogeneity Analysis

Following the classification criteria set by the State Council of China, city sizes are classified according to the resident population (less than 500,000, 500,000 to 1 million, 1 million to 5 million, 5 million to 10 million, and more than 10 million), corresponding to small cities, medium cities, large cities, mega cities and super cities, respectively. To avoid model errors caused by the small sample, we divided the 280 cities in our sample into two categories on this basis, including small cities and large and medium-sized cities (containing the last four classifications). The estimated results are shown in Table 6. The coefficient of DID is positive and statistically significant at 1% in column (1), while it is 0.017 and insignificant at 10% in column (3). Similarly, the coefficient of DID is negative and statistically significant at 1% in column (2), while it is insignificant at 10% in column (4). This suggests that the large and medium-sized cities gain greater economic and environmental gains from NUA policy than smaller cities, implying that the effects of the NUA policy are heterogeneous among the cities of different scales.

Further, according to Chinese geographical divisions, we divided the sample cities into three regions: eastern, central and western. The estimated results are shown in Table 7. The coefficients of DID are positive and statistically significant at 1% in columns (1), (3) and (5), indicating that the NUA policy has a significant positive effect on urban economic growth in all regions. The coefficient of DID is significantly negative at 1% in column (2), significantly negative at 10% in column (4), and negative but insignificant in column (6), indicating that compared to the central and western regions, the curbing effect of the NUA policy on SO_2_ emissions is apparent in the eastern region.

In addition, we use the SDID model according to Equation (2) to reveal the spatial heterogeneity. The estimation results are shown in Table 8. The coefficients of DID are positive and statistically significant in columns (1) and (2) and are negative and statistically significant at 1% in columns (3) and (4), indicating that the effects of the NUA policy still hold after accounting for spatial factors. The spatial coefficient of the W_T,T_D is positive and significant at 5% in columns (1) and (2), while it is significantly negative at 5% in column (3). It can be concluded that the effects of the NUA policy on economic development and environmental pollution have a significant spatial spillover effect in statistics among treated cities. The spatial coefficients of the W_NT,T_D are positive and statistically significant at 5% in columns (1) and (2), but are insignificant in columns (3) and (4). This suggests that the NUA policy promoted economic development in the untreated cities neighboring the treated cities. In addition, the spatial spillover effects of the NUA policy on pollutant emissions diverge significantly between the treated cities and the untreated cities neighboring the treated cities.

## 5. Discussion

To analyze the impact of the NUA policy on economic growth and environmental pollutant emissions, we use the mediation effect test to identify the mechanism of the NUA policy. Based on the above theoretical analysis, we chose green technology innovation (GTEC), industrial structure sophistication (IS) and industrial agglomeration (IA) as intermediary variables and studied the direct or indirect effect of the NUA policy on economic growth and environmental pollutant emissions.

The model setting is as follows:

y_it_ = β_0_ + β_1_DID_it_ + ∑γ_j_x_it_ + μ_i_ + η_t_ + ε_it_ (3)

m_it_ = α_0_ + α_1_DID_it_ + ∑γ_j_x_it_ + μ_i_ + η_t_ + ε_it_ (4)

y_it_ = θ_0_ + θ_1_DID_it_ + θ_2_m_it_ + ∑γ_j_x_it_ + μ_i_ + η_t_ + ε_it_ (5)

where y_it_ represents the dependent variables, including PCGDP and SO_2_ in city i at year t; DID_it_ denotes the dummy variables (NUA_i_ × Time_t_); x denotes the matrix vector of the control variables; and m is the mediating variable, including GTEC, IS and IA. In line with common practice, we use the total number of green technology patents plus one and then take the natural logarithm to measure GTEC. We use the system of equations IS_it_ = ∑r_int_v_int_ to calculate IS, respectively, where i, n and t denote the region, industry and time, respectively, r_int_ is the share of value added to the corresponding industry and v_int_ is the labor productivity of the corresponding industry. We use the locational entropy index to measure the level of urban industrial agglomeration (IA).

Table 9 shows the results for the mediation effect of GTEC. The coefficient of DID is significantly positive at the 5% level in column (1) and significantly negative at the 1% level in column (4), suggesting that the NUA policy has a significant positive impact on economic growth and a significant negative impact on SO_2_, which are consistent with the results in Table 2. The coefficient of DID is 0.179 and significant at the 1% level in column (2), showing that the NUA policy facilitates urban green technology innovation activities. In Table 9, the coefficients of DID and GTEC in column (3) are 0.029 and 0.014, respectively, which are both significant at the 5% level, showing that NUA affects economic growth partly through green technology innovation, supporting hypothesis H2. The coefficients of DID and GTEC in column (6) are −0.200 and −0.053, respectively, which are both significant at the 1% level, suggesting that the green technology innovation plays a significant role in reducing SO_2_ emissions, supporting hypothesis H4.

Table 10 shows the results for the mediation effect of IS. The coefficients of DID and IS in column (3) are 0.031 and 0.006, respectively. Whereas the former is significant at the 5% level, the latter is insignificant at the 10% level, suggesting that the mediating effect of IS is not visible in the economic gains of the NUA policy. The coefficients of DID and IS in column (6) are −0.239 and −0.002, respectively, which are both significant at the 5% level, supporting hypothesis H4. The results show that the NUA policy is conducive to upgrading the industrial structure, prompting the transformation of the city’s industrial structure in the green and low-carbon direction and realizing environmental gains.

Table 11 shows the results for the mediation effect of IA. In Table 11, the coefficient of DID is 0.007 and significant at the 1% level, showing that the NUA policy has a positive effect on urban industrial agglomeration. The coefficients of DID and IA in column (3) are 0.007 and 0.676, respectively, which are both significant at the 5% level, suggesting there exists a partial mediation effect for IA in promoting economic growth, supporting hypothesis H2. The coefficients of DID and IA in column (6) are −0.234 and −0.223, respectively, while the latter is insignificant at the 10% level, showing that the mediation effect of IA is insignificant. In general, the indirect effect of the NUA policy on economic growth is mainly through GTEC and IA, while its indirect effect on environmental pollution emissions is mainly through GTEC and IS.

## 6. Conclusions and Policy Implications

Based on the panel data of 280 cities from 2005 to 2019, this study evaluates the effects of the NUA policy on economic growth and environmental pollution by using the DID model. The main findings are as follows. First, the NUA policy has effectively promoted economic growth while reducing environmental pollutants. Second, we examine the heterogeneity of the NUA policy at the regional and scale levels, showing that the effects of the NUA policy are more evident in the eastern region and in large and medium-sized cities. Furthermore, there is also significant spatial heterogeneity in the spillover effects of NUA policies. Third, the mechanism test showed that the economic and environmental effects of the NUA policy are mainly realized through green technology, industrial agglomeration, and upgrading of industrial structures.

The revelations are as follows. Firstly, regional development planning ensures policy continuity at the macro level and contributes to long-term stable economic development, which is one of the concentrated manifestations of the Chinese model. For developing countries, in particular, it is important to strengthen inter-city linkages and to develop a sustained and coherent regional development policy that plays a role in leading the direction of development and allocating public resources. In addition, the decisive role of the market in resource allocation should be brought into play to promote the full flow of factors. The authorities should break down barriers between cities through institutional reform and give cities of different levels a relatively balanced right to development. According to local conditions, the construction of communities of interest should be promoted in key areas (transport integration, ecological and environmental protection, and industrial docking and collaboration). Secondly, the management should promote the transformation and upgrade of the city’s industrial structure by implementing differentiated industrial policies. Credit and financial incentives should be implemented to attract qualified enterprises and talents. The R&D capability of enterprises can be improved by ensuring the wide application of green technology. Finally, the environmental protection sector should strengthen the synergistic management of environmental pollution emissions between cities, such as further promoting joint prevention and control of air pollution and increasing the comprehensive management of transboundary water pollution. Market-based “green technology + finance” integrated solutions should be explored, and the concept of development of a community of interest should be established.

## Figures and Tables

**Table 1:** Descriptive statistics.

Variable	No. of Obs.	Min.	Mean.	Max.	S.D.
PCGDP	4756	4.595	10.231	15.675	0.849
SO_2_	4562	−1.304	6.773	10.241	1.228
Con	4473	0.0001	0.349	5.671	0.293
GI	4479	0.649	2.198	39.031	1.836
Gov	4479	0.031	0.142	6.041	0.205
Ind	4479	2.660	48.510	90.970	11.022
Fin	4479	0.245	1.144	24.800	1.089
Hum	4452	1.399	3.415	6.895	0.817
Inv	4476	0.087	0.616	10.979	0.648

**Table 2:** Baseline regressive results.

Variables	PCGDP			SO_2_		
	(1)	(2)	(3)	(4)	(5)	(6)
DID	0.068 ** (2.52)	0.028 ** (2.43)	0.024 ** (2.26)	−0.221 *** (−2.82)	−0.233 *** (−3.08)	−0.122 *** (−3.12)
Con		−0.396 *** (−10.64)	−0.337 *** (−10.52)		0.006 (0.04)	0.025 (0.22)
Gi		−0.019 *** (−5.03)	−0.005 (−1.17)		−0.092 ** (−2.44)	−0.035 ** (−2.22)
Gov		−0.106 (−1.43)	−0.570 *** (−5.48)		2.333 *** (2.97)	2.024 *** (5.39)
Ind		0.016 *** (28.89)	0.017 *** (31.66)		0.015 *** (3.62)	0.014 *** (7.40)
Fin		−0.054 *** (−9.72)	−0.036 *** (−7.24)		0.018 (0.61)	0.050 *** (2.82)
Inv		0.090 *** (6.32)	0.125 *** (8.14)		0.030 (0.34)	0.070 (1.26)
City fixed	Yes	Yes	Yes	Yes	Yes	Yes
Time fixed	Yes	Yes	Yes	Yes	Yes	Yes
Observations	4756	4654	3451	4562	4471	3451

Notes: **, and ***, respectively, denote significance levels of 5% and 1%, and the value in brackets is the t value.

**Table 3:** Counterfactual test results.

Variables	PCGDP			SO_2_		
	(1)	(2)	(3)	(4)	(5)	(6)
DID-advance1	0.012 (0.61)			−0.093 * (−1.71)		
DID-advance2		0.001 (0.03)			−0.075 (−1.48)	
DID-advance3			0.007 (−0.41)			−0.073 (−1.52)
Controls	Yes	Yes	Yes	Yes	Yes	Yes
City fixed	Yes	Yes	Yes	Yes	Yes	Yes
Time fixed	Yes	Yes	Yes	Yes	Yes	Yes
Observations	4654	4654	4654	4471	4471	4471

Notes: * denote significance level of 10%, and the value in brackets is the t value.

**Table 4:** Baseline regressive results.

Variables	Employment	Smoke (Dust)	Wastewater
	(1)	(2)	(3)
DID	0.038 *** (2.91)	−0.230 *** (−5.21)	−0.058 * (−1.79)
Controls	Yes	Yes	Yes
City fixed	Yes	Yes	Yes
Time fixed	Yes	Yes	Yes
Observations	4630	4479	4494

Notes: * and ***, respectively, denote significance levels of 10% and 1%, and the value in brackets is the t value.

**Table 5:** Results of endogeneity test.

Variables	PCGDP		SO_2_	
	Phase I	Phase II	Phase I	Phase II
Hist_captial × DID	0.605 *** (42.14)		0.608 *** (43.56)	
DID		0.166 *** (3.79)		−0.961 *** (−8.89)
Controls	Yes	Yes	Yes	Yes
City fixed	Yes	Yes	Yes	Yes
Time fixed	Yes	Yes	Yes	Yes
N	4759	4759	4656	4656
F statistic	225.4		152.93	

Notes: *** denote significance level of 1%, and the value in brackets is the t value.

**Table 6:** The heterogeneity test at the city scale level.

Variables	Large—Medium		Small	
	PCGDP (1)	SO_2_ (2)	PCGDP (3)	SO_2_ (4)
DID	0.053 *** (2.74)	−0.441 *** (−7.01)	0.017 (1.24)	−0.001 (−0.03)
Controls	Yes	Yes	Yes	Yes
City fixed	Yes	Yes	Yes	Yes
Time fixed	Yes	Yes	Yes	Yes
N	4378	4306	4378	4306

Notes: *** denote significance level of 1%, and the value in brackets is the t value.

**Table 7:** The heterogeneity test at the regional level.

Variables	Eastern		Central		Western	
	PCGDP (1)	SO_2_ (2)	PCGDP (3)	SO_2_ (4)	PCGDP (5)	SO_2_ (6)
DID	0.403 *** (6.04)	−0.219 *** (−3.47)	0.335 *** (5.89)	−0.169 * (−1.82)	0.592 *** (5.79)	−0.021 (0.14)
Controls	Yes	Yes	Yes	Yes	Yes	Yes
City fixed	Yes	Yes	Yes	Yes	Yes	Yes
Time fixed	Yes	Yes	Yes	Yes	Yes	Yes
N	4378	4306	4378	4306	4378	4306

Notes: * and ***, respectively, denote significance levels of 10% and 1%, and the value in brackets is the t value.

**Table 8:** The results of spatial regression.

Variables	PCGDP	Employment	SO_2_	Smoke (Dust)
	(1)	(2)	(3)	(4)
DID	0.035 ** (2.08)	0.047 * (1.84)	−0.228 *** (−3.25)	−0.274 *** (−2.96)
W_TT_D	0.380 *** (3.51)	0.297 ** (1.24)	−0.578 *** (−4.50)	−0.463 * (−1.83)
W_NT,T_D	3.742 ** (2.13)	5.016 *** (4.49)	−3.513 (−0.87)	2.157 (−1.54)
Controls	Yes	Yes	Yes	Yes
City fixed	Yes	Yes	Yes	Yes
Time fixed	Yes	Yes	Yes	Yes
N	4654	4654	4471	4471

Notes: *, **, and ***, respectively, denote significance levels of 10%, 5%, and 1%, and the value in brackets is t the value.

**Table 9:** Mediating effect of GTEC.

Variables	PCGDP	GTEC	PCGDP	SO_2_	GTEC	SO_2_
	(1)	(2)	(3)	(4)	(5)	(6)
DID	0.028 ** (2.43)	0.179 *** (4.63)	0.029 *** (2.75)	−0.233 *** (−3.08)	0.179 *** (4.63)	−0.200 *** (−5.45)
GTEC			0.014 ** (2.38)			−0.053 *** (−3.75)
Controls	Yes	Yes	Yes	Yes	Yes	Yes
City fixed	Yes	Yes	Yes	Yes	Yes	Yes
Time fixed	Yes	Yes	Yes	Yes	Yes	Yes
Observations	4271	4271	4271	4212	4271	4212

Notes: ** and ***, respectively, denote significance levels of 5% and 1%, and the value in brackets is the t value.

**Table 10:** Mediating effect of IS.

Variables	PCGDP	IS	PCGDP	SO_2_	IS	SO_2_
	(1)	(2)	(3)	(4)	(5)	(6)
DID	0.028 ** (2.43)	0.232 ** (2.22)	0.031 ** (2.02)	−0.233 *** (−3.08)	0.232 ** (2.22)	−0.239 *** (−3.15)
IS			0.006 (0.97)			−0.002 ** (−2.19)
Controls	Yes	Yes	Yes	Yes	Yes	Yes
City fixed	Yes	Yes	Yes	Yes	Yes	Yes
Time fixed	Yes	Yes	Yes	Yes	Yes	Yes
Observations	4271	4271	4271	4212	4271	4212

Notes: ** and ***, respectively, denote significance levels of 5% and 1%, and the value in brackets is the t value.

**Table 11:** Mediating effect of IA.

Variables	PCGDP	IA	PCGDP	SO_2_	IA	SO_2_
	(1)	(2)	(3)	(4)	(5)	(6)
DID	0.028 ** (2.43)	0.007 *** (4.04)	0.007 ** (2.40)	−0.233 *** (−3.08)	0.007 *** (4.04)	−0.234 *** (−3.09)
IA			0.676 *** (12.27)			−0.223 (−0.40)
Controls	Yes	Yes	Yes	Yes	Yes	Yes
City fixed	Yes	Yes	Yes	Yes	Yes	Yes
Time fixed	Yes	Yes	Yes	Yes	Yes	Yes
Observations	4271	4271	4271	4212	4271	4212

Notes: ** and ***, respectively, denote significance levels of 5%, and 1%, and the value in brackets is the t value.

## Data Availability

Not applicable.

## Appendix A

**Table A1:** The 155 cities in the NUA group.

Urban Agglomerations	City	No. of Cities
Northern Bay	Beihai, Chongzuo, Fangchenggang, Haikou, Maoming, Nanning, Qinzhou, Yangjiang, Yulin, Zhanjiang	10
Chengdu-Chongqing	Chengdu, Dazhou, Deyang, Guang’an, Leshan, Luzhou, Meishan, Mianyang, Nanchong, Neijiang, Suining, Ya’an, Yibin, Chongqing, Ziyang, Zigong	16
Guanzhong Plain	Baoji, Linfen, Pingliang, Qingyang, Shangluo, Tianshui, Tongchuan, Weinan, Xi’an, Xianyang	10
Ha-Chang	Daqing, Harbin, Liaoyuan, Mudanjiang, Qiqihar, Siping, Songyuan, Suihua, Changchun	9
Hubao-Egyu	Baotou, Erdos, Hohho, Yulin	4
Beijing-Tianjin-Hebei	Baoding, Beijing, Cangzhou, Chengde, Hengshui, Langfang, Qinhuangdao, Shijiazhuang, Tangshan, Tianjin, Zhangjiakou	11
Lanxi	Baiyin, Dingxi, Lanzhou	3
South Central Liaoning	Anshan, Benxi, Dalian, Dandong, Fushun, Liaoyang, Panjin, Shenyang, Yingkou	9
The Yangtze River Delta	Anqing, Bengbu, Changzhou, Chizhou, Chuzhou, Hangzhou, Hefei, Huzhou, Jiaxing, Jinhua, Maanshan, Nanjing, Nantong, Ningbo, Shanghai, Shaoxing, Suzhou, Taizhou, Taizhou, Tongling, Wenzhou, Wuxi, Wuhu, Xuancheng, Yancheng, Yangzhou, Zhenjiang, Zhoushan	28
Mid-Yangtze River	Changde, Ezhou, Fuzhou, Hengyang, Huanggang, Huangshi, Ji’an, Jingmen, Jingzhou, Jingdezhen, Jiujiang, Loudi, Nanchang, Pingxiang, Shangrao, Wuhan, Xianning, Xiangtan, Xiangyang, Xiaogan, Xinyu, Yichang, Yichun, Yiyang, Yingtan, Yueyang, Changsha, Zhuzhou	28
Central Plains	Anyang, Bozhou, Fuyang, Handan, Heze, Hebi, Huaibei, Jiaozuo, Jincheng, Kaifeng, Liaocheng, Luoyang, Luohe, Nanyang, Puyang, Sanmenxia, Shangqiu, Suizhou, Xinxiang, Xinyang, Xingtai, Xuchang, Yuncheng, Changzhi, Zhengzhou, Zhoukou, Zhumadian	27

**Table A2:** The 125 cities in the non-NUA group.

Classification	City
Cities in non-NUA group	Huainan, Huangshan, Liuan, Fuzhou, Xiamen, Putian, Sanming, Quanzhou, Zhangzhou, Nanping, Longyan, Ningde, Ganzhou, Zaozhuang, Jining, Tai’an, Laiwu, Linyi, Dezhou, Binzhou, Pingdingshan, Shiyan, Suizhou, Shaoyang, Zhangjiajie, Chenzhou, Yongzhou, Huaihua, Guangzhou, Shaoguan, Shenzhen, Zhuhai, Shantou, Foshan, Jiangmen, Zhaoqing, Huizhou, Meizhou, Shanwei, Heyuan, Qingyuan, Dongguan, Zhongshan, Chaozhou, Jieyang, Yunfu, Liuzhou, Guilin, Wuzhou, Guigang, Baise, Hezhou, Hechi, Laibin, Sanya, Panzhihua, Guangyuan, Bazhong, Guiyang, Liupanshui, Zunyi, Anshun, Kunming, Qujing, Yuxi, Baoshan, Zhaotong, Lijiang, Lincang, Yan’an, Hanzhong, Ankang, Jiayuguan, Jinchang, Wuwei, Zhangye, Jiuquan, Xining, Yinchuan, Shizuishan, Wuzhong, Urumqi, Karamay, Baicheng, Guyuan, Jinan, Qingdao, Zibo, Dongying, Yantai, Weifang, Weihai, Rizhao, Taiyuan, Datong, Shuo Yangquan, Shuozhou, Jinzhong, Xinzhou, Lvliang, Wuhai, Chifeng, Tongliao, Bayannur, Ulanqab, Jinzhou, Fuxin, Tieling, Chaoyang, Huludao, Tonghua, Baishan, Jixi, Hegang, Shuangyashan, Yichun, Jiamusi, Qitaihe, Heihe, Xuzhou, Lianyungang, Huaian, Suqian, Quzhou, Lishui
